# Preparation of a Polymeric Phosphoramide Flame-Retardant and Its Effect on the Flame-Retardant Properties of Epoxy Resin

**DOI:** 10.3390/polym16091224

**Published:** 2024-04-27

**Authors:** Hao Wang, Yinjie Wang, Yan Su, Chuang Yu, Jia Han, Jiping Liu

**Affiliations:** School of Materials Science and Engineering, Beijing Institute of Technology, 5 Zhongguancun South Street, Haidian District, Beijing 100081, China; wanghao000723@163.com (H.W.); suyan126abc@126.com (Y.S.); yuchuang95@sina.com (C.Y.); hanjia89yiyi@sina.cn (J.H.)

**Keywords:** epoxy resin, polymeric, curing agent, flame-retardant

## Abstract

The flammability of epoxy resins and knowing how to achieve curing are particularly important factors during use. A novel approach for enhancing the fire resistance and reducing the smoke emission of epoxy resin during the curing process is suggested, which involves the utilization of a three-source integrated polymerization intumescent flame-retardant. In this study, the synthesis of poly 4,4-diaminodiphenylsulfone spirocyclic pentaerythritol bisphosphonate (PCS) is achieved through using solution polymerization, utilizing 4,4′-diaminodiphenylsulfone (DDS) and spirocyclic pentaerythritol bisphosphorate disphosphoryl chloride (SPDPC) as initial components. Following that, the EP underwent the inclusion of PCS to examine its resistance to heat, its ability to prevent flames, its effectiveness in reducing smoke and its curing effect. Compared to the unmodified epoxy resin, the addition of PCS can not only cure the epoxy resin, but also decompose before the epoxy resin and has a good carbonization effect. With the addition of 7 wt.% PCS, the LOI value can achieve 31.2% and successfully pass the UL-94 test with a V-0 rating. Moreover, the cone calorimeter experiment demonstrated a noteworthy decline of 59.7% in the maximum heat release rate (pHRR), 63.7% in overall heat release (THR), and 42.3% in total smoke generation (TSP). Based on the examination of TG-FTIR and SEM findings, there is ample evidence to suggest that PCS, functioning as a phosphorus-nitrogen intumescent flame-retardant that combines three origins, has the potential to exhibit a favorable flame-retardant impact in both its gas and condensed phases.

## 1. Introduction

With the development of polymer research, epoxy resins, particularly bisphenol epoxy resins, have found extensive applications in aviation, renewable energy, adhesives, electronic devices, and various other industries, owing to their exceptional mechanical characteristics, adhesion, and the ease of machining [1,2,3,4]. The global demand for EPs is projected to keep increasing due to the expansion of industries like transportation, aerospace, marine coatings, and composite materials [5,6,7]. However, epoxy resin is easy to burn in the air and emits a large amount of smoke and droplets [8,9], thereby posing challenges in meeting the aforementioned development criteria. Hence, there is a pressing requirement to enhance the fire-resistant properties of epoxy resin.

At present, the more efficient halogen-based flame retardants are criticized for their high harm to the environment [10,11,12]. Therefore, it is urgent to explore green and efficient flame retardants. The rise of intumescent flame retardants (IFRs) offers fresh opportunities for the advancement of environmentally friendly and effective flame retardants, and they are widely seen as one of the most promising alternatives to traditional halogen flame retardants. In theory, the introduction of the P element in flame retardants can not only promote the formation of a carbon layer, but can also effectively capture oxygen-free radicals and reduce oxygen concentration. The intervention of the N element produces a large number of non-combustible gas bodies in the combustion process. The synergistic effect of P and N can achieve an excellent gas phase and a condensed phase flame-retardant effect [13,14,15]. Many studies have confirmed the practicability of phosphorus-nitrogen flame-retardant polymer materials [16,17,18]. These characteristics, such as being free of halogens, emitting low smoke, having low toxicity, and being anti-dripping, align with the future direction of environmental protection [19,20].

Implementing the use of one-component IFR as a new strategy, this integrated method effectively reduces the use of flame retardants, while greatly ensuring the original performance of the material [21,22]. SPDPC, functioning as a cage compound containing both an acid source and a carbonization agent, facilitates the conversion of oxygen-containing compounds into carbon through dehydration during combustion. Furthermore, the presence of active P-Cl bonds on both ends of SPDPC renders it a highly suitable precursor for the synthesis of intumescent flame retardants [23,24,25,26,27].

Over the last couple of years, it has been demonstrated that polymer resins can benefit greatly from the use of spiro-ring structure compounds as highly efficient flame retardants [28,29]. The intumescent flame-retardant PDSPB, prepared by Ma, greatly solved the problem of difficult dispersion of nanomaterials in the polymer matrix and provided flame retardancy for nanocomposites [30]. Zhang created a new flame-retardant—called BSPB—by combining phosphorus and nitrogen. This was then used as a chain extender to chemically bond with a waterborne polyurethane (WPU) skeleton. The result was a range of flame-retardant waterborne polyurethane (FRWPU) materials that successfully prevented the spread of heat and flames within the polyurethane upon exposure to fire [31]. Based on the synthesis of SPDPC, Song further synthesized small molecule flame-retardant PIPC and polymeric flame-retardant SAPC using a two-step method. Both of them can achieve a better flame-retardant effect within 10% of the addition amount, and both of them have an excellent compatibility with epoxy resin and have long-term practicability [32,33]. However, the above studies all need to add curing agents to prepare liquid EP in the required flame-retardant sample form, which increases the possibility of EP performance degradation to a certain extent [7,34].

Based on the existing research, no matter the use of traditional flame retardants or the flame retardants in the IFRs system based on SPDPC, EP materials need to add a large number of curing agents to achieve curing molding after adding flame retardants. At the same time, we found that the flame retardants in the existing research often require a long response time to achieve a flame-retardant effect, and there are problems in that flame retardants are sometimes easy to precipitate and lose their flame-retardant properties. Because of the above problems, we prepared a polymeric flame-retardant containing a secondary amine group by using the active hydrogen reaction between the double-active end group of SPDPC and the amino group in the curing agent DDS, so that it has both curing and flame-retardant characteristics. At the same time, it can achieve a fast, lasting, and effective response that is not easy to precipitate, and a good smoke suppression effect in epoxy resin.

## 2. Materials and Methods

### 2.1. Materials

Tianjin Nankai Yungong Synthetic Technology Co., Ltd., situated in Tianjin, China, supplied pentaerythritol (PER) with a minimum purity of 98%, phosphorus oxychloride (POCl_3_) with a minimum purity of 98%, aluminum trichloride (AlCl_3_) of analytical reagent grade, and glacial acetic acid with a purity of 98%. Beijing Chemical Plant, based in Beijing, China, provided acetonitrile (AR), triethylamine (TEA) with a purity of 99.5%, and 4,4-diaminodiphenyl sulfone (DDS) with a minimum purity of 98%. Anhydrous ethanol, dichloromethane, dimethyl sulfoxide, and acetone were supplied by Weiss Chemical Reagent Co., Ltd. (AR, Beijing, China). The epoxy resin bisphenol A (E-44, with an epoxy equivalent of 0.44 mol/100 g) was obtained from Synthetic Material Co., Ltd., located in Yueyang, Hunan, China. Before usage, it is necessary to distill phosphorus oxychloride and acetonitrile again.

### 2.2. Synthesis of PCS

**Scheme 1:**
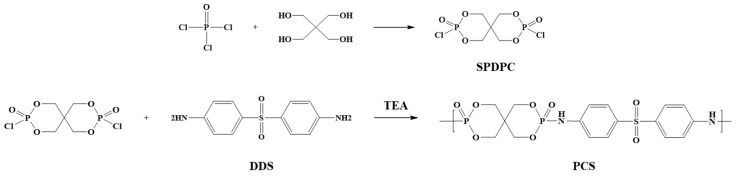
Preparation of SPDPC and PCS.

The preparation of PCS was divided into two steps (Scheme 1). To begin, the first action was to combine the thoroughly crushed PER (6.8 g; 0.05 mol) and AlCl_3_ (0.72 g) with the POCl_3_ (23.1 g; 0.15 mol) obtained via distillation. After stirring for 1 h at room temperature under nitrogen, the mixture was slowly heated to 80 °C and was left to react until no HCl gas was detected. After the reaction, the unrefined substance was rinsed with dichloromethane and acetone three times consecutively. Afterwards, we purified the product by further removing the unreacted pentaerythritol using glacial acetic acid. The high-purity intermediate SPDPC was obtained with a yield of 91% after vacuum drying.

In the next phase, at room temperature and in the presence of nitrogen, DDS (5.3 g; 0.021 mol) and SPDPC (5.9 g; 0.02 mol) were scattered in 40 mL acetonitrile for 1 h. Subsequently, TEA (4.04 g; 0.04 mol) was dripped into the reaction mixture by funnel, and then the temperature was gradually raised to 80 °C while stirring for a duration of 10 h. Upon the completion of the reaction, the solvent was removed via vacuum distillation to obtain the raw material. Then, it was dissolved in anhydrous ethanol and purified by reverse precipitation with dimethyl sulfoxide and dichloromethane. After vacuum drying the product to a constant weight, 65.1% of the purified product was obtained.

### 2.3. Preparation of Composites EP/PCS

**Scheme 2:**
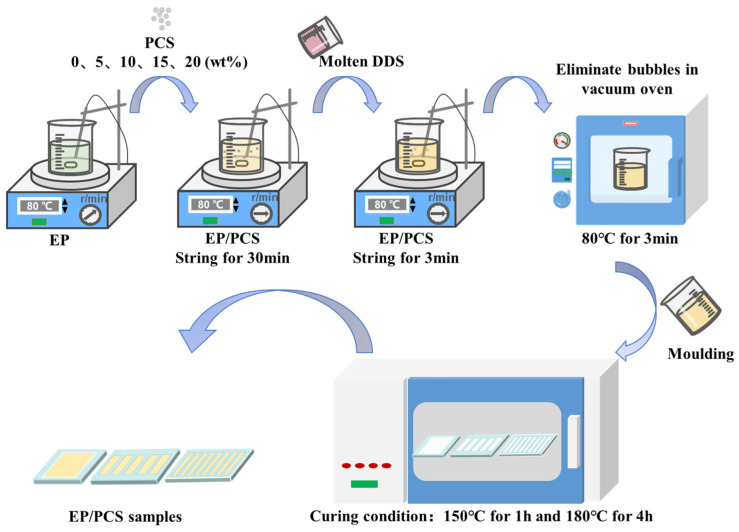
Fabrication process of the EP/PCS composites.

Epoxy resin composites with different mass fractions were prepared according to the following process (Scheme 2). To begin, we incorporated PCS into the preheated and evenly stirred epoxy resin, followed by stirring at a temperature of 80 °C until the absence of any visible particles (with the increase in PCS dosage, this time needs to be extended, generally 1~2 h). Afterwards, DDS was added to the mixture mentioned earlier and agitated for a duration of 3 min (The curing agent for the epoxy resin was always maintained at 1:5. With the addition of PCS as a substitute for the curing agent, we reduced the use of the equal quality curing agent. When the addition of PCS was 20 wt.%., DDS was no longer added.). Afterwards, the beaker was moved to the preheated vacuum oven to remove all bubbles (This stage was usually 3~5 min, otherwise the viscosity of the product increased for too long and was not easy to pour out). Following this, it was promptly injected into the preheated mold. Afterwards, the blend was treated at a temperature of 150 °C for a duration of 1 h and was subsequently raised to 180 °C for a period of 4 h. The table labeled Table 1 provides the ratio of the EP composite formulations. We found that PCS can complete the curing of EP within the curing time and the curing effect is good, which does not affect the subsequent flame-retardant performance tests.

### 2.4. Characterizations

Fourier transform infrared (FTIR) spectroscopy was performed using the Bruker Optics TENSOR 27 FTIR spectrometer (Bruker Optics, Beijing, China).

Nuclear Magnetic Resonance (NMR) experiments were performed using the FT-80A NMR spectrometer from Varian, located in Beijing, China. The solvent used was DMSO-d6, and both ^1^H and ^31^P were included in the experiments.

Thermogravimetric analysis (TGA) was conducted using the TOLEDO STARE thermal analyzer (Mettler-Toledo, Zurich, Switzerland). The test conditions were a heating rate of 20 °C/min, nitrogen or air atmosphere, gas flow rate of 50 mL/min, and test range of 50~600 °C for PCS and 50~700 °C for EP/PCS composites.

The LOI was examined using the JF-3 oxygen index device (Nanjing, China) from Nanjing Jiangning Analytical Instrument Co., Ltd., following the ASTM D 2863-2008 standard. The dimensions of the sample were 130 mm in length, 6.5 mm in width, and 3 mm in height.

A CZF-5 horizontal vertical burning tester manufactured by Phoenix Instruments Co., Ltd. (Suzhou, China) was used to perform vertical burning tests according to the UL-94 standard. The dimensions of the sample were 130 mm in length, 13 mm in width, and 3 mm in thickness.

The Fire Testing Technology apparatus (Phoenix Instruments Co., Ltd., Suzhou, China) was utilized for conducting cone calorimeter measurements. The measurements followed the ISO 5660 protocol and were performed with an incident radiant flux of 50 kW/m^2^. The dimensions of the sample were 100 mm by 100 mm by 3 mm.

For scanning electron microscopy (SEM), the Hitachi S4800 (Hitachi Limited, Tokyo, Japan) was employed at an accelerating voltage of 15 kV.

To perform TG-IR, the thermalgravimetric analyzer TGA/DSC-1 from Mettler-Toledo (Zurich, Switzerland) and the Fourier transform infrared spectroscopy Bruker Tensor 27 were linked using a heating transmission pipe.

## 3. Results

### 3.1. Characterization of PCS

**Figure 1:**
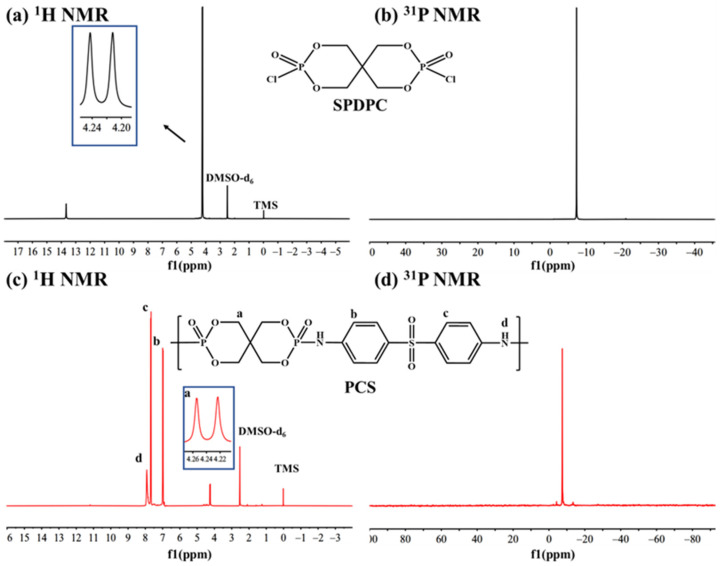
The SPDPC spectra were acquired through ^1^H NMR (**a**) and ^31^P NMR (**b**), whereas the PCS spectra were obtained via ^1^H NMR (**c**) and ^31^P NMR (**d**).

The NMR spectra of SPDPC and PCS, which includes ^1^H NMR and ^31^P NMR, are shown in Figure 1. The spiro structure’s successful synthesis of the intermediate SPDPC was preliminarily proven by the two peaks of equal height at 4.21 and 4.24 ppm (Figure 1a), indicating the presence of methylene. According to Figure 1b, the phosphorus peak in SPDPC was at −7.6 ppm, providing additional evidence of the excellent symmetry and singular composition of SPDPC [33]. In the case of PCS, as depicted in the ^1^H NMR (Figure 1c), the preservation of methylene double peaks at 4.22 and 4.25 ppm, the emergence of two distinct protons at 6.9–7.8 ppm denotes the aromatic composition, and the resonance peak at 8.0 ppm was attributed to N–H. Furthermore, the ^31^P NMR spectrum (Figure 1d) of PCS exhibited a striking resemblance to SPDPC, indicating that the phosphorus atoms share an identical chemical surrounding. Consequently, the aforementioned analysis provides the initial verification of the accomplished synthesis of PCS.

**Figure 2:**
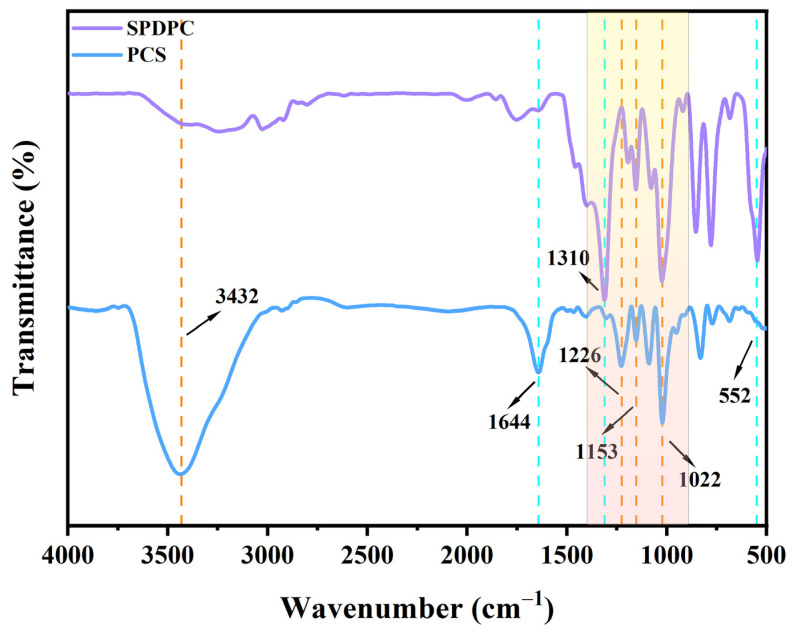
FTIR spectra of the SPDPC and PCS.

The infrared spectra of the intermediates SPDPC and PCS are shown in Figure 2. The disappearance of the characteristic P-Cl peak at 552 cm^−1^ and the appearance of the typical -NH peak at 3440 cm^−1^ indicated that the reaction was successful. Additionally, the spikes at 1022 cm^−1^ and 1310 cm^−1^ correspond to the P=O and P-O-C vibrational absorption spikes in the spiro configuration, respectively. Moreover, the preservation of the skeletal feature peak of the benzene ring at 1644 cm^−1^ and the stretching vibration peak of the S=O at 1226 cm^−1^ indicates that the DDS structure in the PCS remained intact. The molecular structure can be confirmed and the successful preparation of the target product PCS can be proven through these distinct absorption peaks.

**Figure 3:**
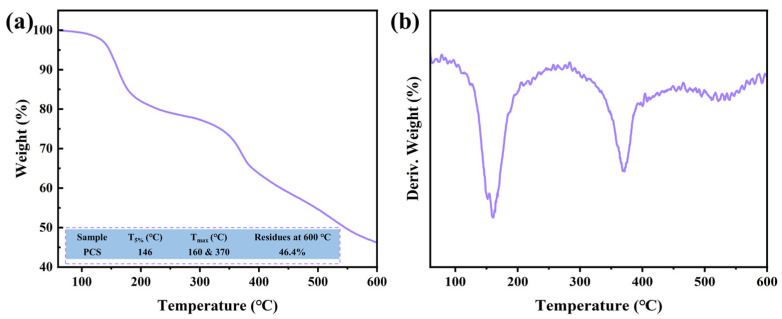
(**a**) TGA and (**b**) DTG under the nitrogen atmosphere of the PCS.

The thermal stability of PCS is shown in Figure 3. The TGA curve (Figure 3a) shows that PCS has a char residue of up to 46.4% at 600 °C, indicating that PCS has a good charring ability. The results of DTG (Figure 3b) showed that PCS mainly had two thermo-gravimetric stages. The first thermo-gravimetric stage occurred at about 160 °C, corresponding to the breaking of the phosphamide bond. The second thermo-gravimetric stage occurred at about 370 °C, corresponding to the formation stage of carbon residue. In addition, its initial thermal decomposition temperature is lower than EP (T_5%_ = 146 °C), indicating that PCS can be degraded before the decomposition of the substrate, thus displaying a flame-retardant effect before the polymer is completely burned.

### 3.2. Thermal Properties and Fire Safety of EP/PCS Composites

**Figure 4:**
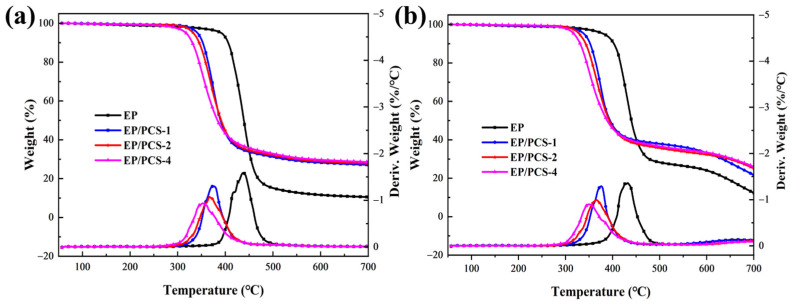
Nitrogen was used to obtain the TGA and DTG profiles. (**b**) Air was used to obtain the TGA and DTG profiles.

Under the nitrogen atmosphere, the thermal degradation of pure EP is comparable to EP/PCS, with only a single degradation process occurring (Figure 4a). Pure EP was degraded in the range of 380 °C to 450 °C, accompanied by the maximum thermal degradation rate at 440 °C, indicating that the degradation of the EP main chain mainly occurred at this stage. The introduction of PCS made the T_onset_ and T_max_ of EP/PCS composites earlier than that of pure EP, respectively (Table 2). Notably, Figure 4a and Table 2 demonstrate a considerably elevated carbon residue in EP/PCS-2 and EP/PCS-4 specimens in comparison to the pure EP. The presence of the phosphoramide structure in PCS is responsible for this. On the one side, it decreases the speed of pyrolysis for the carbon framework and the creation of flammable volatile tiny compounds. On the other hand, by generating functional structures such as C=N or P-N, it provides additional interchain covalent bonds and catalyzes the cross-linking of skeletal carbon to form a stable reticular carbon layer, thereby promoting the increase in carbon content to play a flame-retardant role [35].

In contrast to the nitrogen-induced degradation process observed in EP composites, both pure EP and EP/PCS exhibited a two-step degradation pattern when exposed to air. The initial stage, occurring between 320 °C and 400 °C, primarily involved the decomposition of the phosphamide group found in the flame-retardant PCS. The subsequent stage, which took place between 550 °C and 700 °C, primarily involved the pyrolysis of alkyl chains and aromatic structures. The T_onset_ and T_max_ of the pure EP and EP/PCS have a certain degree of advance, and the carbon residue of the sample at 700 °C is lower than that in nitrogen. It is important to mention that Figure 4b and Table 2 reveal that the carbon residue values of EP/PCS-2 and EP/PCS-4 samples are significantly greater than those of pure EP. This suggests that the inclusion of PCS enhances the thermal stability of the EP composites when exposed to high temperatures. Simultaneously, PCS and EP exhibit a robust interplay during thermal degradation, resulting in the development of an expanded carbon layer that safeguards the underlying polymer matrix, effectively functioning as a proficient flame-retardant.

**Figure 5:**
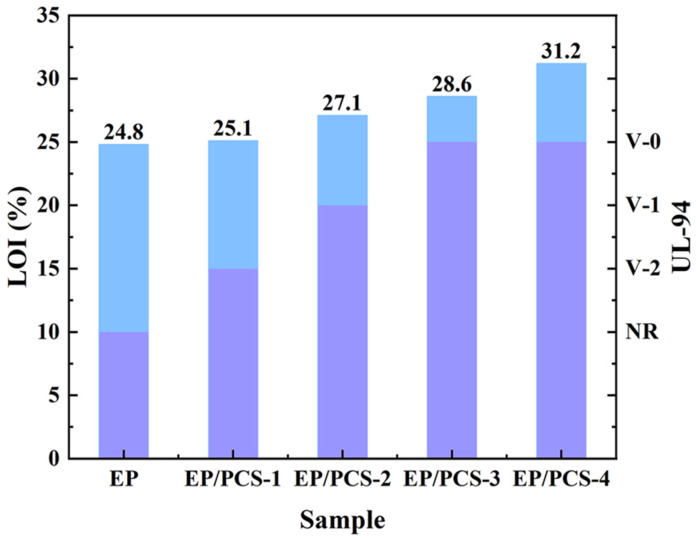
LOI values and UL-94 rating of the EP composites.

The fire safety features of epoxy resin were evaluated by assessing the flame-retardant properties of PCS through UL-94, LOI, and cone calorimetry tests. By incorporating PCS, the epoxy resin composites exhibit enhanced LOI and UL-94 values without any occurrence of the droplet phenomenon (as shown in Figure 5 and Table 3). This shows that PCS can inhibit the dripping of EP and avoid the possibility of causing the combustion of other polymers. When the addition of PCS is 20%, the LOI value can reach 31.2%, and the UL-94 can reach the V-0 level. The data above validate that PCS can effectively enhance the flame retardancy of epoxy resin, preventing the potential for igniting other polymers and increasing the risk of fire.

**Figure 6:**
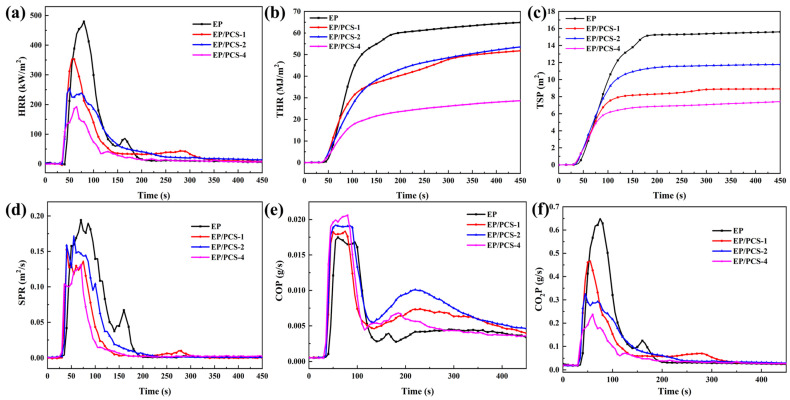
(**a**) HRR, (**b**) THR, (**c**) TSP, (**d**) SPR, (**e**) COP (CO production), and (**f**) CO_2_P (CO_2_ production) curves for all EP composites.

The samples underwent cone calorimetry testing to further assess their detailed combustion characteristics. The test data of pure EP, EP/PCS-2, and EP/PCS-4 samples were selected and summarized as follows (Figure 6 and Table 4). The inclusion of 20 wt% PCS resulted in a maximum decrease of 59.7% in the pHRR compared to the EP control. Additionally, there was a maximum decrease of 63.7% in THR (at 200 s) and a maximum decrease of 42.3% in TSP. The inclusion of 20 weight percent PCS led to a significant reduction in the pHRR of epoxy composites, bringing it down from 959 to 386 kW/m^2^. This decrease emphasizes the notable decline in the fire risk of epoxy composites during real fire situations, establishing it as a crucial factor for evaluating fire safety. Moreover, the ignition time of EP/PCS sample happens earlier in comparison to the pure EP, due to the rapid increase in the surface temperature of EP when mixed with PCS at the beginning of the heating phase. This outcome aligns with the findings from the thermogravimetric analysis of PCS, suggesting that PCS can enhance flame retardancy at a quicker rate. The unstable P-O-C bond and P-N-C bond in the structure are degraded first, and acid substances such as phosphoric acid or pyrophosphoric acid are produced [36,37].To some degree, these acids facilitate the additional degradation of the specimen to a certain extent and significantly enhance the creation of a carbon layer on the surface of EP/PCS materials. In addition, with the addition of PCS, the CO and CO_2_ produced by the epoxy resin during the combustion process are reduced, indicating that the EP sample added with PCS only partially degraded during the combustion process. With the addition of PCS, the CO and CO_2_ produced by the epoxy resin during the combustion process are reduced. This is mainly because the phosphoric acid, metaphosphoric acid, and other substances produced by the early degradation of PCS catalyze the degradation of EP into carbon, isolate oxygen, and inhibit heat transfer, thereby reducing the production of combustible gas [38]. We know that at the scene of the fire, smoke, high heat and other fuel combustion caused by dripping are the main factors affecting the rescue, so the combustion characteristics of the PCS added above provide a guarantee for fire safety.

### 3.3. Analysis of EP/PCS Composites in Condensed and Gaseous Phases

**Figure 7:**
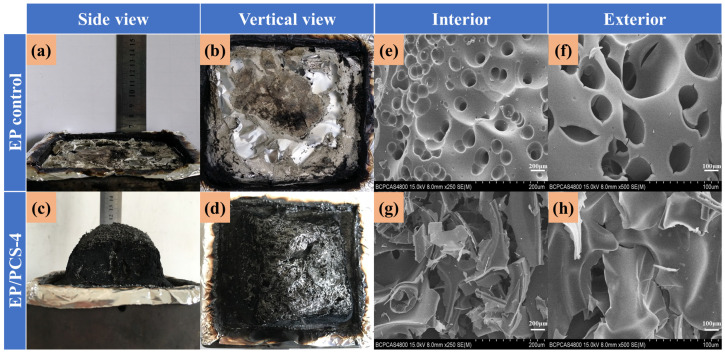
Digital photos (**a**–**d**) and SEM images (**e**–**h**) of the char residues of the EP composites.

In order to showcase the influence of the formed intumescent char layer on the burning properties of the EP/PCS samples and acquire an additional understanding of their flame-retardant mechanism, the digital camera and scanning electron microscope were utilized to capture the residue that remained after combustion (Figure 7). The pure EP burns more fully, and only a small amount of carbon residue is left in the aluminum foil (Figure 7a,b). The combustion of EP/PCS-4 (Figure 7c,d) clearly demonstrates a denser and higher carbon residue compared to pure EP, suggesting that PCS enhances the cross-linking of EP into carbon.

The SEM morphology of char residue is depicted in Figure 7e–h. The residual carbon of pure EP cannot form a continuous carbon layer during combustion. There are many pore structures on the surface of internal and external residual carbon, which promotes oxygen flow and heat transfer so that pure EP material will burn violently in a short time (Figure 7e,f). Additionally, the combustion of pure EP material leaves minimal residue, aligning with the findings of the thermogravimetric analysis. The internal and external residual carbon of EP/PCS-4 samples after adding 20 wt.% PCS will form a dense lamellar structure. For the internal residual carbon, it not only has a dense lamellar structure but also has some bubble structure, which is attributed to the decomposition of PCS to produce non-combustible gases and the migration of phosphate derivatives to the surface (Figure 7g). Furthermore, because of the migration of phosphoric acid derivatives generated from the breakdown of PCS during combustion, the outer residual carbon exhibits a higher density compared to the residual carbon present inside (refer to Figure 7h). Significantly, in the compact state, PCS, a curing agent modified by a three-source DDS with fire-resistant characteristics, can effectively impart flame-retardant properties to EP composites.

**Figure 8:**
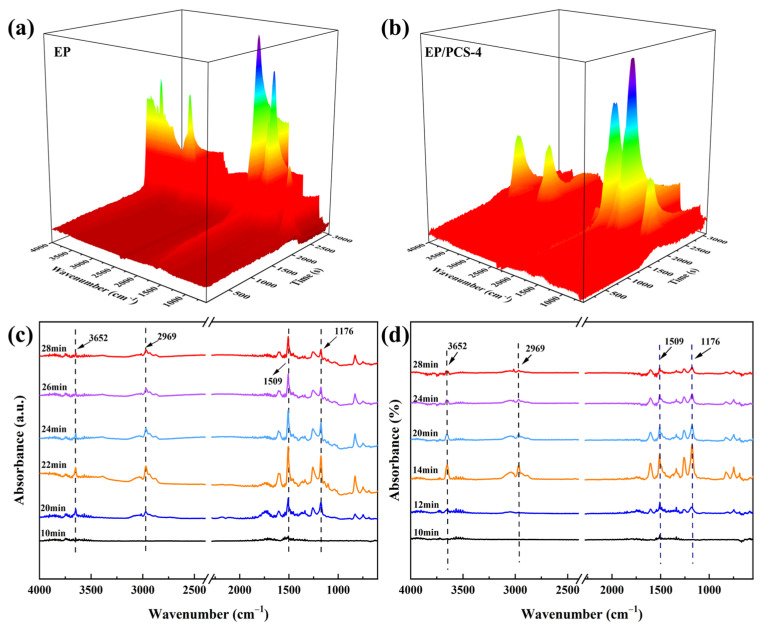
(**a**,**b**) EP and EP/PCS-4 3D TG-FTIR spectra, (**c**) EP FITR spectra at various time intervals, (**d**) EP/PCS-4 FTIR spectra at different time intervals.

**Figure 9:**
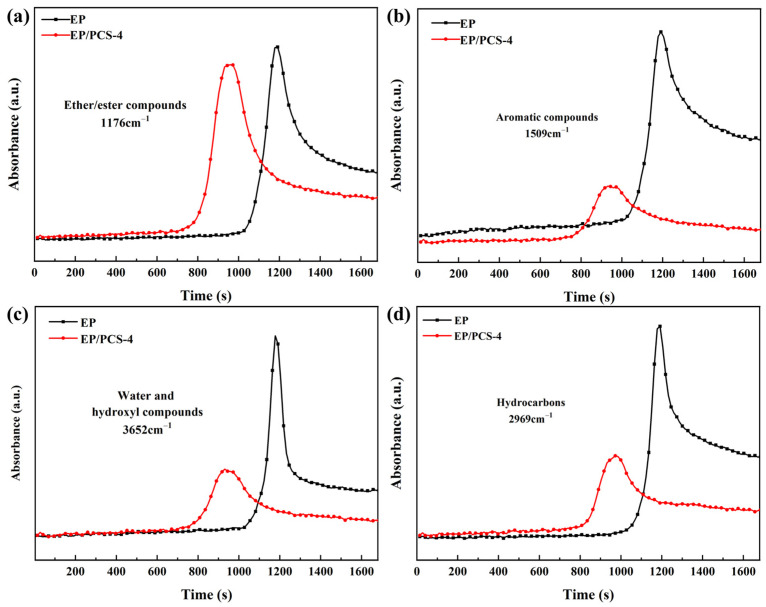
(**a**–**d**) ester compounds, aromatic compounds, hydrocarbons and water release profiles for EP and EP/PCS-4.

Flame-retardant substances in the gas phase also play a major role in preventing combustion and reducing thermal feedback in materials that can catch fire. Figure 8a,b visually depicts the gas phase degradation process of the EP control and EP/PCS-4, which was examined by comparing the 3D TG-FTIR spectra. The infrared spectra of gas products in different periods were selected from the TG-FTIR combined data to further analyze the gas phase products in the combustion process (Figure 8c,d). The primary information regarding the degradation products of EP/PCS-4 in the gas phase appears to be identical to that of pure EP. These primarily consist of water and phenols (3652 cm^−1^), hydrocarbons (2950~2880 cm^−1^), compounds containing ether/ester (1263 cm^−1^, 1176 cm^−1^), and aromatic compounds (1602 cm^−1^, 1509 cm^−1^, 1342 cm^−1^) [39,40]. Nevertheless, the primary release period of the primary deteriorated substances of EP/PCS-4 occurred approximately 8 min prior, suggesting that PCS has the ability to enhance the thermal deterioration of EP at reduced temperatures. To further examine the disparity between the pure EP and EP/PCS samples, Figure 9 presents a comparative chart illustrating the primary deteriorations of the pure EP and EP/PCS-4 samples. The degradation of EP/PCS-4 occurs earlier, and the primary degradation byproducts of EP/PCS-4 are less than those of EP, particularly H_2_O, aromatic compounds, and hydrocarbons. This further demonstrates that PCS has the ability to catalyze the carbonization of the EP system at a lower temperature and decrease the emission of flammable gases, thereby providing flame-retardant properties. Based on the above analysis, PCS can play the role of priority response and has a flame-retardant effect that is consistent with expectations.

### 3.4. The Mechanism That Makes EP/PCS Composites Resistant to Flame

**Scheme 3:**
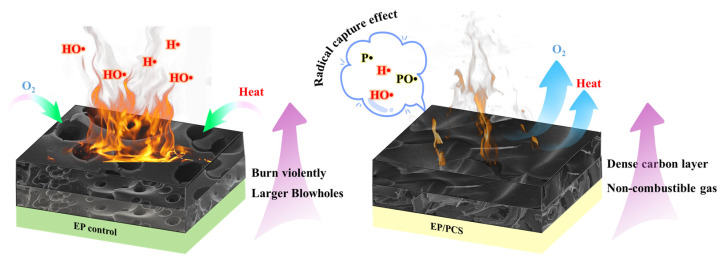
Schematic of the flame-retardant mechanism.

The deduction of the flame-resistant mechanism for the EP/PCS produced in this study was determined in the following manner (Scheme 3). PCS, classified as a phosphorus-nitrogen intumescent flame-retardant, holds significance in enhancing flame retardancy in both the gas and condensed phases, as indicated by the preceding analysis. During the condensed phase, the phosphorus-based substances generated through PCS decomposition act as catalysts for the creation of a carbon layer that is resistant to high temperatures. Moreover, the compounds produced through PCS pyrolysis contributes to the creation of a continuous lamellar structure within the melted carbon residue, serving as a safeguarding shield that hinders the inward flow of heat and oxygen. In the gaseous state, phosphorus-containing radicals generated from PCS decomposition capture active radicals (H•, HO•) on the side of the polymer surface, leading to a quenching effect [41]. At the same time, in the combustion process, PCS restricts the generation of flammable gas while enhancing the emission of non-flammable gas during EP decomposition, leading to asphyxiation and dilution effects. In summary, PCS acts as a flame-retardant curing agent that combines the following factors: its ability to expand, capture free radicals, dilute oxygen concentration, and catalyze charring. These properties effectively contribute to flame retardancy and smoke suppression when EP undergoes combustion.

## 4. Conclusions

In this work, based on curing agent DDS, a polymerized intumescent flame-retardant PCS with both curing and flame-retardant properties was designed and synthesized, which made the epoxy composite material have better fire safety performance. Some interesting new findings and perspectives are concluded here:

1. In the epoxy resin with only PCS flame-retardant, curing at 150 °C for one hour and curing at 180 °C for 4 h can achieve the effect of curing epoxy resin with the traditional curing agent DDS. The existence of PCS can be decomposed before EP, which has a good effect and greatly prevents the further severe combustion of EP. By combining three sources, the carbon chain structure of PCS can serve as a carbon source for carbon formation, the P group as an acid source, and the N group as a gas source. This integration enables the achievement of flame retardancy in both the condensed and gas phases. PCS with both flame-retardant and curing provides a new reference for the development of flame retardants due to its numerous functionalities.
2. The EP composites, containing 20 weight percent PCS, exhibited an LOI of 31.2% and successfully cleared the UL-94 examination, receiving a V-0 level. Moreover, with the TTI being advanced, the pHRR, THR, and TSP values for EP/PCS were obviously reduced. With the addition of flame-retardant PCS, we achieved the pHRR reduction of 59.7%, the THR reduction of 63.7%, and the TSP reduction of 42.3%, which is extremely important for flame-retardant polymer materials and suppression of smoke emissions.
3. We found that the addition of PCS increased the thickness of the carbon layer in the combustion process of EP by nearly 5 cm, indicating that this flame-retardant can give full play to the effect of the IFRs. TG-FTIR and SEM results further confirmed that PCS can generate a dense and continuously expanded carbon layer with a stable structure after heating, isolating the transfer of oxygen and heat, and generating a non-combustible gas to dilute oxygen, with excellent flame-retardant performance.
4. Although this work has prepared a new type of flame-retardant with both flame-retardant and curing effects, this paper only elaborates on the improvement of flame-retardant performance. The preparation of multifunctional polymer materials should be the focus of our attention. In addition, there are still many possibilities for the preparation of new flame retardants. We should explore the synthesis of more efficient and environmentally friendly flame retardants to achieve our goals while ensuring economic costs.

## Figures and Tables

**Table 1:** Formulations for the EP composites.

Samples	EP (wt.%)	PCS (wt.%)	DDS (wt.%)
EP-0	100	0	20
EP/PCS-1	95	5	15
EP/PCS-2	90	10	10
EP/PCS-3	85	15	5
EP/PCS-4	80	20	0

**Table 2:** Thermal degradation properties of the EP composites.

Samples	T_onset_ (°C)		T_max_ (°C)		Residual Char (500 °C, wt.%)		Residual Char (700 °C, wt.%)	
	N_2_	Air	N_2_	Air	N_2_	Air	N_2_	Air
EP-0	388	367	440	430	15	28	10	12
EP/PCS-2	317	307	366	365	32	36	28	25
EP/PCS-4	296	290	351	349	33	37	29	26

**Table 3:** LOI and Vertical combustion tests data.

Samples	LOI (%)	UL-94	t_1_ (s)	t_2_ (s)	Dripping
EP-0	24.8	NR	-	-	Yes
EP/PCS-1	25.1	V-2	23.1	8.9	No
EP/PCS-2	27.1	V-1	15.2	7.3	No
EP/PCS-3	28.6	V-0	4.2	3.5	No
EP/PCS-4	31.2	V-0	3.1	1.9	No

**Table 4:** Cone analysis of the EP/PCS composites.

Samples	TTI ^1^ (s)	pHRR (kW/m^2^)	THR (MJ/m^2^)	TSP (m^2^)	Mean COY (g/s)	Mean CO_2_P (g/s)
EP-0	45	959	66.3	15.78	0.116	2.17
EP/PCS-2	38	506	53.4	11.72	0.108	1.71
EP/PCS-4	31	386	24.1	7.01	0.104	0.96

^1^ TTI: time to ignition.

## Data Availability

Data are contained within the article.
